# Temporal patterns of influenza a subtypes and B lineages across age in a subtropical city, during pre-pandemic, pandemic, and post-pandemic seasons

**DOI:** 10.1186/s12879-019-3689-9

**Published:** 2019-01-25

**Authors:** Linlin Zhou, Huiping Yang, Yu Kuang, Tianshu Li, Jianan Xu, Shuang Li, Ting Huang, Chuan Wang, Wanyi Li, Mingyuan Li, Shusen He, Ming Pan

**Affiliations:** 1 Department of Microbiology, West China School of Basic Medical Sciences & Forensic Medicine, Sichuan University, Chengdu, 610041 China; 2 Sichuan Center for Disease Control and Prevention, No. 6 Zhongxue Road, Wuhou District, Chengdu, 610041 Sichuan People’s Republic of China; 3 Department of Medical Technology, West China School of Public Health, Sichuan University, Chengdu, 610041 China

**Keywords:** Influenza A subtypes, Influenza B lineages, Epidemiology, Southwestern China, Seasonality, Pandemic, Age distribution

## Abstract

**Background:**

Seasonal patterns of influenza A subtypes and B lineages in tropical/subtropical regions across age have remained to be explored. The impact of the 2009 H1N1 pandemic on seasonal influenza activity have not been well understood.

**Methods:**

Based on a national sentinel hospital-based influenza surveillance system, the epidemiology of influenza virus during 2006/07–2015/16 was characterized in the subtropical city, Chengdu. Chengdu is one of the most populous cities in southwestern China, where the first reported case of A/H1N1pdm09 in mainland China was identified. Wavelet analysis was applied to identify the periodicities of A/H3N2, seasonal A/H1N1, A/H1N1pdm09, Victoria, and Yamagata across age, respectively. The persistence and age distribution patterns were described during the pre-pandemic (2006/07–2008/09), pandemic (2009/10), and post-pandemic (2010/11–2015/16) seasons.

**Results:**

A total of 10,981 respiratory specimens were collected, of which 2516 influenza cases were identified. Periodicity transition from semi-annual cycles to an annual cycle was observed for composite influenza virus as well as A/H3N2 along in Chengdu since the 2009 H1N1 pandemic. Semi-annual cycles of composite influenza virus and A/H3N2 along were observed again during 2014/15–2015/16, coinciding with the emergence and predominance of A/H3N2 significant antigenic drift groups. However, A/H1N1pdm09, Victoria, and Yamagata generally demonstrated an annual winter-spring peak in non-pandemic seasons. Along with periodicity transitions, age groups with higher positive rates shifted from school-aged children and adults to adults and the elderly for A/H1N1pdm09 during 2009/10–2010/11 and for A/H3N2 during 2014/15–2015/16.

**Conclusions:**

Differences in periodicity and age distribution by subtype/lineage and by season highlight the importance of increasing year-round influenza surveillance and developing subtype/lineage- and age-specific prevention and control measures. Changes of periodicity and age shifts should be considered in public health response to influenza pandemics and epidemics. In addition, it is suggested to use quadrivalent influenza vaccines to provide protection against both influenza B lineages.

**Electronic supplementary material:**

The online version of this article (10.1186/s12879-019-3689-9) contains supplementary material, which is available to authorized users.

## Background

Influenza can cause epidemics and pandemics which give rise to significant morbidity and mortality worldwide [1, 2]. Viruses of influenza A subtypes and B lineages are the main causes of the disease burden in humans.

Despite the mass of research on seasonal influenza, seasonality of influenza virus has not been well characterized in tropical/subtropical regions. Diverse seasonal patterns, including annual and semi-annual cycles, as well as year-round activity have been widely observed [3–5]. In addition, the epidemic peaks of type A and B viruses were found to coincide less frequently in tropical/subtropical countries than in temperate countries [6, 7]. However, epidemiological studies focusing on individual influenza subtypes/lineages are limited. Consequently, analyzing surveillance data with greater spatial resolution (i.e., at the city level rather than on a national scale) of specific influenza types, subtypes, and lineages in tropical/subtropical regions is crucial to develop local prevention and control measures, as well as to understand influenza persistence worldwide.

During March and early April 2009, the 2009 pandemic influenza A virus subtype H1N1 (A/H1N1pdm09) emerged in the Americas and subsequently caused the 2009 H1N1 pandemic worldwide [8, 9]. After the pandemic, A/H1N1pdm09 replaced the pre-existing seasonal influenza A virus subtype H1N1 (seasonal A/H1N1), circulating as a seasonal influenza virus with influenza A virus subtype H3N2 (A/H3N2) and influenza B lineages, B/Victoria/2/87-like (Victoria) and B/Yamagata/16/88-like (Yamagata) [10, 11]. It is suggested that novel antigenic variants might interrupt the circulation of the resident viruses, possibly through competition for susceptible hosts [12]. However, the impact of the newly emerged pandemic strain on the seasonal epidemics of the resident influenza subtypes/lineages has not been well understood. The epidemiological characteristics of A/H1N1pdm09 during the post-pandemic periods remain to be explored.

This retrospective study aimed to describe and compare the epidemiological and virological characteristics of influenza A subtypes and B lineages during pre-pandemic, pandemic, and post-pandemic seasons and to reveal the impact of the 2009 H1N1 pandemic on influenza activity in a subtropical city in detail. Influenza surveillance data during 2006/07–2015/16 were collected in the subtropical city, Chengdu, where the first reported case of A/H1N1pdm09 in mainland China was identified [13]. Serving as the capital of Sichuan province, Chengdu is one of the most populous cities in southwestern China and is the western center of logistics, commerce, and finance. Despite the importance of Chengdu, to our knowledge, there is little research available on the epidemiological characteristics of influenza virus in this city. Based on ten-year influenza surveillance the seasonality and the age distribution of A/H3N2, seasonal A/H1N1, A/H1N1pdm09, Victoria, and Yamagata were identified. The persistence of specific/individual influenza subtypes/lineages in each respiratory season was evaluated.

## Materials and methods

### Influenza surveillance data

Chengdu is located at the 30.67°N and 104.07°E, with a registered population of 14.7 million [14]. It has a typical subtropical humid climate. A sentinel hospital-based influenza surveillance system was initiated in 2001 by the Sichuan Center for Disease Control and Prevention, as part of the National Influenza Surveillance Network in China [15]. Since 2006, the sentinel hospitals have started to report the year-round surveillance data on a centralized online system maintained by the Chinese Centre for Disease Control and Prevention, which has significantly decreased the missing data rate. Therefore, we focused on 2006/07–2015/16 season in this study.

During 2006/07–2007/08, influenza surveillance was conducted in two sentinel hospitals. Since 2008/09, one more sentinel hospital was included in the surveillance system. All three sentinel hospitals are Grade III level A (top-class in China). The number of physicians and nurses remained at 17, 30, and 38 in these hospitals respectively, in order to keep consistency during the ten-year surveillance. Each week, sentinel hospitals reported the numbers of total visits to outpatient and/or emergency departments as well as patients with influenza-like-illness (ILI) to a centralized online system maintained by the Chinese Center for Diseases Control and Prevention. ILI was defined by the World Health Organization [WHO] as sudden onset of fever with measured body temperature ≥ 38 °C and a cough or sore throat in the absence of other diagnoses. In each sentinel hospital, the first one or two (the first three or four since 2013) nasopharyngeal swabs were collected on each day of the weeks in which ILI data were recorded, resulting in 10–15 (20–30 since 2013) specimens per hospital per surveillance week. Clinical specimens were inoculated in Madin-Darby canine kidney (MDCK) cells and/or specific pathogen free (SPF) chicken embryos. Hemagglutination inhibition (HI) test and/or real-time reverse transcription PCR (RT-PCR) assay were performed to identify the types, subtypes, and lineages of influenza viruses. ILI consultation and influenza virological surveillance were carried out following the guideline for national influenza surveillance [16] and more details could be found in the previous description and study in China [33].

### Influenza periodicity and seasonality analyses

A(H1N1)pdm09 emerged in Chengdu in May 2009, therefore, a respiratory season was defined as the period from May of one year to April of the following year in this study. Three pre-pandemic seasons (2006/07–2008/09), the pandemic season (2009/10), and six post-pandemic seasons (2010/11–2015/16) were included. In 2009/10, 31 (3.6%) influenza A virus-positive isolates were unsubtyped amd were allocated among three subtypes, A/H3N2, seasonal A/H1N1, and A/H1N1pdm09, according to the seasonal ratio of total numbers for each subtype. Influenza B virus-positive specimens that were not determined were similarly allocated into two lineages, Victoria, and Yamagata, except in 2009/10 when 78 out of 92 (84.8%) influenza B virus-positive specimens were not determined.

Time series of the monthly influenza virus positive rates which were calculated as monthly numbers of positive specimens (all influenza viruses, or each A subtype, or each B lineage) divided by monthly total numbers of specimens tested were compiled. Since individual move from one age group to another, age in relation to birth year was inspected. Monthly age-specific positive rates of each subtype/lineage were aggregated into four age groups, 0–4, 5–17, 18–59, and ≥ 60 years. To characterize influenza virus seasonality, wavelet analysis was applied. Wavelet analysis has been used for various infectious diseases data [17, 18] to identify periodicities in time series data and help track variations in periodicity over time [19]. The sowas package [20, 21] implemented in MATLAB R2016b (Mathworks, Natick, MA) was applied to conduct wavelet analysis. Time series data were square root-transformed prior to analysis as previously applied to other epidemiological analyses [22–24] in order to stabilize variance because of the increased number of specimens collected and the 2009 H1N1 pandemic.

### Statistical analyses

To assess differences in age distribution of each subtype/lineage, the positive rates of a given subtype/lineage across four age groups for a given respiratory season were compared using chi-square or Fisher’s exact tests. A *P* value satisfying *P* <  0.05 was considered as statistically significant. Statistical analyses were performed using SPSS v21 (SPSS Inc., Chicago, IL, USA).

## Results

### Seasonality and periodicity of influenza virus in Chengdu

**Fig. 1:**
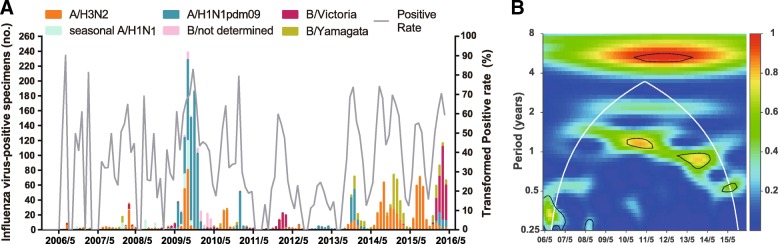
Composite influenza virus activity in Chengdu, China, 2006/07–2015/16. **a** The monthly positive rates of all influenza viruses combined and monthly distribution of influenza A subtypes (A/H3N2, seasonal A/H1N1, and A/H1N1pdm09) and B lineages (Victoria and Yamagata). **b** Wavelet power spectrum of the monthly positive rates of all influenza viruses combined. Black lines highlight periodicities that reach statistical significance of 95% based on 1000 Monte Carlo simulation. The region outside the white-curved cone indicate the presence of edge effects. The power values were shown in the panel on the right. Time series have been square-root transformed

During 2006/07–2015/16, 645,205 (0.85%) ILI cases were recorded. A total of 10,981 respiratory specimens were detected and 2516 (22.91%) influenza viruses were isolated (Table 1). The composite influenza virus activity including all influenza isolates exhibited changes in periodicity. During the pre-pandemic seasons 2006/07–2008/09 and the post-pandemic seasons 2014/15–2015/16, semi-annual cycles were identified, with one peak in summer or early autumn and the other in winter or early spring. However, an annual cycle with a peak in winter or early spring was detected during 2011/12–2013/14 (Fig. 1). Wavelet analysis showed that the semi-annual cycle was statistically significant (*P* <  0.05) in 2014/15–2015/16, and the annual cycle was statistically significant (*P* <  0.05) in 2010/11 and 2013/14 (Fig. 1b).

**Table 1:** Number, detection rates and proportions of influenza virus by age group in Chengdu, 2006/07–2015/16

			No. (frequency^1^) of influenza A subtypes and B lineages								Proportion^2^ of influenza A subtypes and B lineages					
Age group^3^	Specimens, No.	Positive Specimens, No. (frequency)	A	A/H3N2	Seasonal A/H1N1^4^	A/H1N1pdm09^5^	B	Victoria	Yamagata	A	A/H3N2	Seasonal A/H1N1	A/H1N1pdm09	B	Victoria	Yamagata
Total	10,981	2516 (22.9%)	1776 (16.2%)	803 (7.3%)	111 (1.0%)	862 (7.8%)	736 (6.7%)	333 (3.0%)	325 (3.0%)	70.6%	31.9%	4.4%	34.3%	29.3%	13.2%	12.9%
0–4	3298	347 (10.5%)	228 (6.9%)	158 (4.8%)	13 (0.4%)	57 (1.7%)	118 (3.6%)	54 (1.6%)	53 (1.6%)	65.7%	45.5%	3.7%	16.4%	34.0%	15.6%	15.3%
5–17	3337	1109 (33.2%)	725 (21.7%)	282 (8.5%)	51 (1.5%)	392 (11.7%)	384 (11.5%)	168 (5.0%)	177 (5.3%)	65.4%	25.4%	4.6%	35.3%	34.6%	15.1%	16.0%
18–59	3936	995 (25.3%)	773 (19.6%)	328 (8.3%)	46 (1.2%)	399 (10.1%)	220 (5.6%)	104 (2.6%)	88 (2.2%)	77.7%	33.0%	4.6%	40.1%	22.1%	10.5%	8.8%
≥60	410	65 (15.9%)	50 (12.2%)	35 (8.5%)	1 (0.2%)	14 (3.4%)	14 (3.4%)	7 (1.7%)	7 (1.7%)	76.9%	53.8%	1.5%	21.5%	21.5%	10.8%	10.8%
*P* value^6^		< 0.001	< 0.001	< 0.001	< 0.001	< 0.001	< 0.001	< 0.001	< 0.001							

*Abbreviation*: *NA* not available

^a^The age-specific positive rates of laboratory-confirmed influenza cases

^b^The proportion of subtype- or lineage-positive cases within each age group

^3^ 11 influenza B isolates within 0–4 age group were not characterized into lineages and one isolate was of a mixed infection; 39 influenza B isolates within 5–17 age group were not characterized into lineages; 28 influenza B isolates within 18–59 age group were not characterized into lineages and two isolates were of mixed infections; one isolate was of a mixed infection within ≥60 age group

^4^ Seasonal A/H1N1 viruses were isolated from 2006/07 to 2009/10

^5^ A/H1N1pdm09 viruses were isolated since 2009/10

^6^ *P* value of chi-square tests for positive rates of laboratory-confirmed influenza cases across four age groups. Fisher exact test was used instead when cell counts were lower than five

**Fig. 2:**
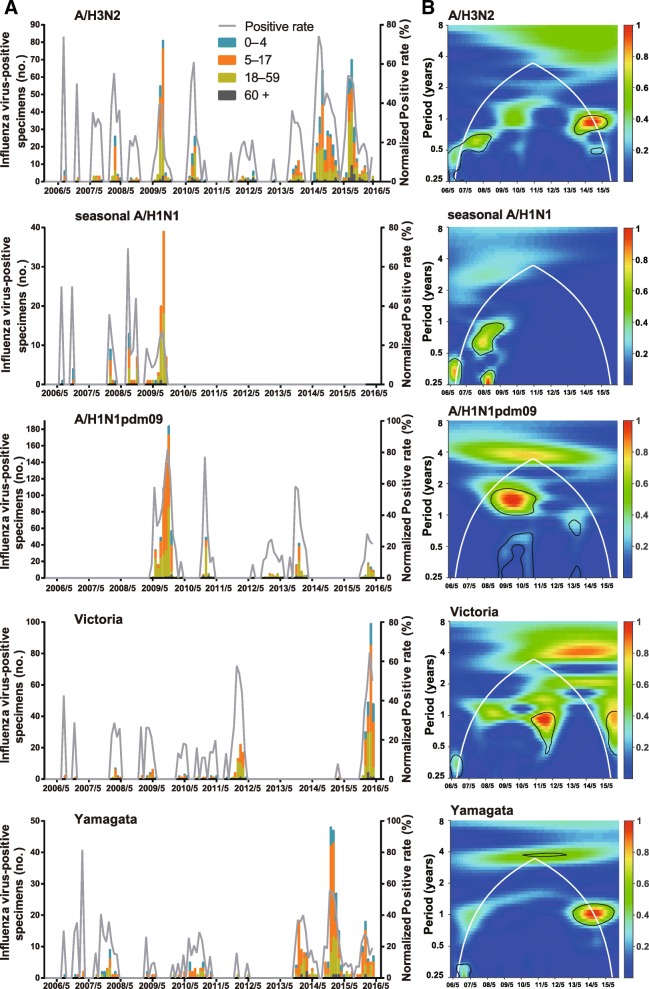
Seasonal distribution and wavelet power spectrum of influenza subtypes/lineages. **a** The monthly positive rates of influenza A subtypes (A/H3N2, seasonal A/H1N1, and A/H1N1pdm09) and B lineages (Victoria and Yamagata) and monthly numbers of laboratory confirmed cases by age group. **b** Wavelet power spectrum of the monthly positive rates of influenza subtypes/lineages. Black lines highlight periodicities that reach statistical significance of 95% based on 1000 Monte Carlo simulation. The region outside the white-curved cone indicate the presence of edge effects. The power values were shown in the panel on the right. Time series have been square-root transformed

To understand seasonality of each subtype/lineage across age in Chengdu, 803, 111, 862, 333, and 325 influenza cases of A/H3N2, seasonal A/H1N1, A/H1N1pdm09, Victoria, and Yamagata were analyzed (Table 1). Individual subtypes and lineages demonstrated annual and/or semi-annual cycles (Fig. 2 and Additional file 1: Figure S1). Before the pandemic, A/H3N2 exhibited semi-annual cycles (*P* <  0.05), with one peak during August–September and the other during January–February. Similar to composite influenza activity, an annual cycle with a peak in August–September was observed for A/H3N2 since 2009/10. Based on wavelet analysis, A/H3N2 did not exhibit any statistically significant periodicity during 2011/12–2012/13 when A/H3N2 viruses were not frequently detected. In addition to the major summer-autumn peak, a minor winter peak of A/H3N2 resurged resulting in the co-existence of statistically significant (*P* <  0.05) semi-annual cycles and the annual cycle during 2014/15–2015/16. Both annual and semi-annual cycles were observed for seasonal A/H1N1 in 2006/07–2008/09. Additional surveillance data might be necessary to identify the epidemic pattern. Seasonal A/H1N1 completely disappeared from the Chengdu population after the pandemic. A/H1N1pdm09 emerged in Chengdu in May 2009 and demonstrated a minor summer peak and a major winter peak during 2009/10. After the pandemic, an annual cycle with a peak in December–February predominated. Both Victoria and Yamagata exhibited an annual cycle (*P* <  0.05) with a winter–spring peak in most seasons.

All four age groups demonstrated similar seasonal patterns for influenza subtypes/lineages, although a few exceptions were observed (Additional file 1: Figure S1). For example, statistically significant semi-annual cycles (*P* <  0.05) of A/H3N2 were observed only in school-aged children in 2006/07, as well as in adults and the elderly in 2009/10 and 2014/15–2015/16. Moreover, instead of an annual cycle, statistically significant (*P* <  0.05) semi-annual cycles were observed in the elderly in 2013/14 for A/H1N1pdm09. In addition, influenza viruses were not detected in the elderly during the pre-pandemic seasons. Therefore wavelet analysis was unable to identify any statistically significant influenza periodicity for this age group.

### Subtype/lineage distributions by season

On average, 70.6 and 29.3% of influenza-positive cases during the 10 surveillance seasons were type A and B influenza viruses, respectively (Table 1). Compared with those in the pre-pandemic period, a statistically significant decrease of positive rates in the pandemic was observed for most non-pandemic influenza viruses, except for A/H3N2 with similar positive rates between pre-pandemic and pandemic period. After the pandemic, seasonal A/H1N1 completely disappeared from the Chengdu population, and the positive rate of A/H1N1pdm09 became significantly lower than in the pandemic (Additional file 2: Table S1). Positive rates of A/H3N2 and Yamagata during the post-pandemic seasons were similar with those during the pre-pandemic seasons, while the positive rates of Victoria in the post-pandemic seasons were significantly higher than in the pre-pandemic seasons. A type or subtype/lineage was considered ‘dominant’ in Chengdu if it account for 50% or more of the total influenza isolates for a particular respiratory season. The predominant influenza subtypes/lineages observed in Chengdu are shown in Fig. 1a and Additional file 2: Table S1. There were at least two subtypes/lineages co-circulating in each season. One or two influenza A subtypes predominated in most seasons, except in 2011/12 when Victoria predominated. Influenza A subtypes generally co-circulated in most seasons, but A/H1N1pdm09 viruses were not detected in 2011/12 and 2014/15. Two influenza B lineages co-circulated during most seasons, except in 2012/13–2013/14 when Victoria viruses were not detected.

### Heterogeneous age distributions of individual subtypes/lineages

**Fig. 3:**
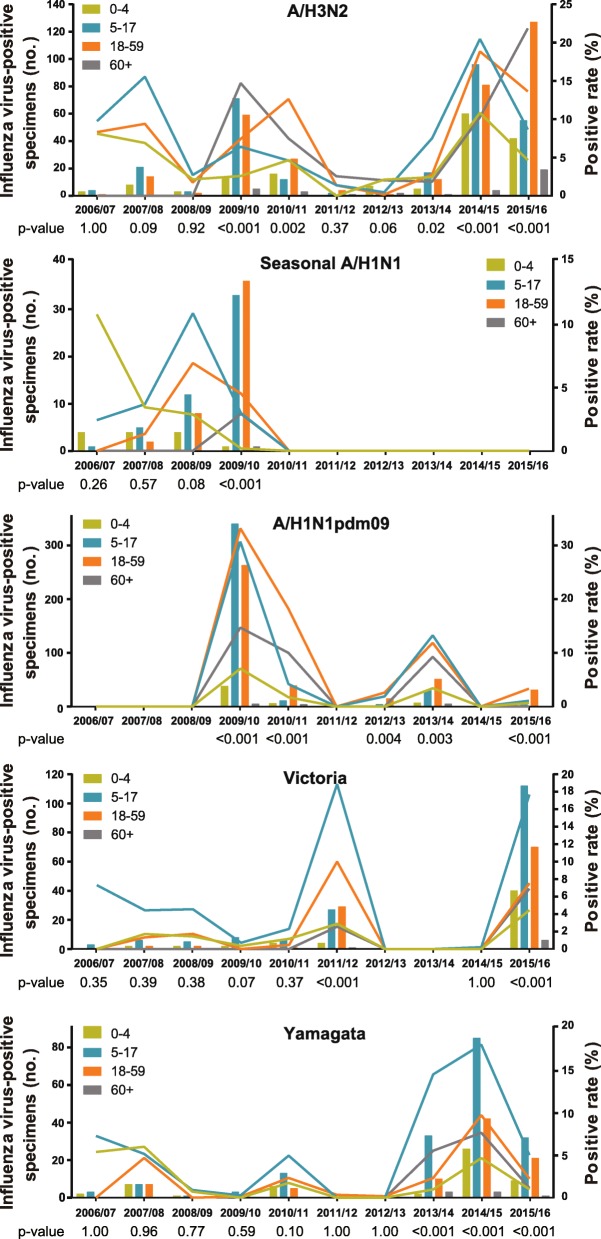
Age distribution of influenza subtypes/lineages. Laboratory-confirmed cases of influenza A subtypes (seasonal A/H1N1, A/H1N1pdm09, and A/H3N2) and influenza B lineages (Victoria and Yamagata) in Chengdu during 2006/07–2015/16. *P* values of chi-square tests for positive rates of each subtype/lineage across age groups in each respiratory season were listed. Fisher’s exact test was used instead of the chi-square test when seasonal counts were less than five

The positive rates of all influenza viruses combined over the ten surveillance years exhibited statistically significant difference across four age groups, 0–4, 5–17, 18–59, and 60+ years. The highest positive rate was observed in school-aged children for all influenza viruses combined as well as for each subtype/lineage (Table 1). However, when assessed by respiratory season, the age distribution patterns varied between subtypes/lineages (Fig. 3).

Prior to the pandemic, there were not any statistically significant difference between seasonal positive rates of influenza subtypes and lineages (Fig. 3). During the pandemic season, the lowest positive rates of A/H3N2 and seasonal A/H1N1 were found in children aged 0–4 years, while A/H1N1pdm09 were more frequently isolated in school-aged children and adults than in other age groups. In 2010/11 the age groups with higher seasonal positive rates for A/H1N1pdm09 changed to adults and the elderly. In the subsequent seasons, A/H1N1pdm09 appeared to be more frequently detected in school-aged children or adults. The highest seasonal positive rates of A/H3N2 were not consistently detected in any specific age group during the post-pandemic seasons. Notably, similar to A/H1N1pdm09, age shift behavoir was also observed for A/H3N2. A/H3N2 were more frequently isolated in school-aged children and adults in 2014/15. However, age groups with higher positive rates changed to adults and the elderly in 2015/16. Lastly, both Victoria and Yamagata were more frequently detected in school-aged children.

## Discussion

### Seasonality and periodicity of influenza virus in Chengdu

10-year surveillance showed periodicity transitions of composite influenza virus activity in Chengdu, a subtropical city in southwestern China. Composite influenza activity changed from semi-annual cycles during pre-pandemic seasons to annual cycles since the 2009 pandemic. Moreover, semi-annual cycles were observed again during 2014/15–2015/16 when A/H3N2 major antigenic drift groups, A/Switzerland/9715293/2013-like (SW13) and A/HongKong/5738/2014-like (HK14) [25], predominated. These A/H3N2 strains dominated in 2014/15 in many countries and caused massive morbidity and mortality because of the antigenic variation [26]. This seasonal pattern was different from the temperate regions and many tropical/subtropical areas with well-defined annual or semi-annual peaks [3–5]. Virological data of influenza virus demonstrated that an annual cycle with one peak in winter was noticeable in the temperate region of China, including Beijing and Shandong province [27, 28]. Influenza virus peaked in summer in Guangdong province in subtropical southern China [29]. However, seasonal pattern was not determined in Chongqing which is adjacent to Sichuan province [30]. A number of factors might drive influenza circulation patterns, including viral evolution, host susceptibility, demographic characteristics, and environmental conditions. An epidemiological study during the pre-pandemic period in Hong Kong found non-stationary seasonality of composite influenza virus activity, which might be caused by a lack of herd immunity when significant antigenic changes in A/H3N2 emerged and predominated [31]. However, the seasonality and interaction of individual influenza subtypes/lineages remain to be investigated.

By analyzing the time series of individual influenza A subtypes and B lineages across age, the differences in seasonality among influenza subtypes/lineages and the different response of resident subtypes/lineages when a novel pandemic strain or new antigenic groups emerged and predominated were observed. Our study firstly found periodicity transitions of A/H3N2, which were similar to composite influenza activity. Semi-annual periodicity of A/H3N2 shifted to an annual cycle since the 2009 pandemic and became evident again coinciding with the emergence and predominance of A/H3N2 major antigenic drifts in 2014/15–2015/16. The periodicity transmissions of A/H3N2 have not been reported previously and are possibly related to the emergence and predominance of the novel pandemic subtype or major antigenic drifts in a largely susceptible population. Secondly, after the pandemic, the pre-existing seasonal A/H1N1 was replaced by A/H1N1pdm09 as observed in other studies [12, 22]. Thirdly, A/H1N1pdm09 generally exhibited an annual cycle peaked in winter or early spring except for 2011/12 and 2014/15 when this virus subtype was not detected. ‘Skip-and-resurgence’ behavior of A/H1N1pdm09 was found during 2011/12–2012/13 in Europe and Eastern Asia, which might be related to the level of immunity as well as the number of susceptible individuals in the population [32]. Our study found that the ‘skip-and-resurgence’ behavior recurred during 2014/15–2015/16. It has just been less than ten years since the emergence of A/H1N1pdm09. Therefore, it is not known whether more ‘skip-and-resurgence’ processes would occur in the future. Fourthly, both Victoria and Yamagata exhibited an annual cycle with a winter-spring peak, which was consistent with previous studies of combined influenza B lineages across China [33]. In addtion, our study showed that the annual cycle of both Victoria and Yamagata did not change according to the significant antigenic variations of influenza A viruses during the pandemic and post-pandemic seasons.

In general, the co-circulation and interaction of A/H3N2, seasonal A/H1N1, A/H1N1pdm09, Victoria and Yamagata constituted the composite influenza activity. The mechanism of the discrepant seasonality has not yet been clarified. But viral evolution, host susceptibility, and environmental factors might drive influenza circulation activity [3, 4, 34, 35]. The extent of periodicity transitions might be associated with the genetic differences and degrees of the antigenic change of the novel pandemic subtype or major antigenic variants, the antigenic relatedness of resident influenza viruses, and the level of host herd immunity. Moreover, similar seasonal patterns of influenza subtypes/lineages across age were identified, which might indicate that the factors driving influenza circulation are probably the same across different age groups.

### Heterogeneous age distributions of influenza subtypes/lineages

Our study found that the highest positive rate of each subtype/lineage was in school-aged children for all study seasons combined. However, age distribution patterns varied by subtype/lineage and by season. Interestingly, age groups with higher positive rates for A/H3N2 shifted from school-aged children and adults in 2014/15 to adults and the elderly in 2015/16. The same age shift behavior of A/H3N2 has not been reported elsewhere but was observed for A/H1N1pdm09 from the 2009/10 pandemic season to 2010/11 in this study and some previous studies [12, 36]. School-aged children and working adults have high social contact rates, so they are likely to experience the highest incidence of infection during the initial phase of a pandemic in a susceptible population [37, 38]. The age shift of A/H1N1pdm09 was proposed to be due to the age heterogeneity in the pre-existing immunity obtained from the first wave of the pandemic [35, 37]. It is noted that the age shift of A/H3N2 occurred when A/H3N2 major antigenic drift groups, SW13 and HK14, emerged and predominated in the Chengdu population during 2014/15–2015/16. Therefore, the same scenario explaining the age shift of A/H1N1pdm09 might also be applicable for A/H3N2.

### Implication for influenza prevention and control measures

Understanding the epidemiological characteristics of influenza virus are critical for directing prevention and control measures. Since school-aged children and working adults were likely to have highest detection rates of infection for the emergence of novel pandemic strains or major antigenic drifts, it might be beneficial to prioritize influenza vaccination and implement interventions (e.g., school-closure) for school-aged children and working adults to prevent and control influenza outbreaks and pandemics in Chengdu [39, 40]. Influenza vaccine has been introduced in China since 1998 [41], but the immunization program has not been carried out. There is growing interest in establishing routine immunization programs nationwide [42]. Currently, only trivalent inactivated influenza vaccine (TIV) is available in the market in China. Every year, TIV with updated vaccine strains recommendated by the WHO for the influenza season, generally from November of one year to April of the following year, in the northern hemisphere is applied in Chengdu. Differences in seasonality of influenza subtypes/lineages would complicate the optimization of vaccination campaigns. Therefore, except for following the timing of vaccination which is typically recommended for the Northern Hemisphere in winter, A/H3N2 activity should be accommodated. The timing of vaccination recommendated by the WHO for the influenza season in the southern hemisphere, generally from May to October of one year, might be incorporated. In addition, our study found that the two influenza B lineages co-circulated during most seasons. Accordingly, it is suggested that quadrivalent influenza vaccine (QIV) instead of TIV should be used to provide protection against both influenza B lineages.

Our study has some limitations that might motivate future research. Firstly, our virological surveillance data focused only on patients with ILI from sentinel hospitals, which might be improved by selecting a random sample of people with influenza-like symptoms from both hospitals and the community. Secondly, during the ten-year surveillance, only a subset of ILI cases were tested, typed, and subtyped, which might get some age groups over sampled and might not represent all of the ILI cases. To minimize the potential bias, the first one or two (the first three or four since 2013) nasopharyngeal swabs on each day of the weeks were collected as previously described [33]. Moreover, the sampling strategy might have a minor impact on our study because we used the detection rates but not the number of positive cases of subtypes/lineages to assess the pattern of influenza activity as previously described [12, 40]. We used the detection rates of influenza virus instead of ILI cases to describe influenza activity due to the following reasons. Firstly, other respiratory viruses, like human rhinovirus, human parainfluenza virus, respiratory syncytial virus, human adenovirus, and so on, might cause clinical symptoms similar to influenza [43]. Therefore, the characteristics of ILI data might reduce the reliability for describing the pattern of influenza epidemics. Secondly, the ILI data are not able to reveal influenza activity of different types and subtypes/lineages. Lastly, our data only cover three pre-pandemic and six post-pandemic influenza seasons. On-going surveillance is necessary to reveal age-specific influenza seasonality after the pandemic.

## Conclusion

This study broadens our understanding of the epidemiological and virological characteristics of specific influenza subtypes/lineages across age groups during pre-pandemic, pandemic, and post-pandemic seasons in the subtropics. The co-circulation of A/H3N2, seasonal A/H1N1, A/H1N1pdm09, Victoria, and Yamagata together constituted the composite influenza activity. Differences in periodicity and age distribution patterns by subtype/lineage and by season was identified. Our findings highlight the importance of strengthening year-round influenza surveillance and developing age-specific prevention and control measures, with due consideration of summer-autumn epidemics, periodicity transition, and age shift behavoir. In addition, a quadrivalent influenza vaccine is recommend to provide protection against both influenza B lineages.

## Additional files

Additional file 1: **Figure S1.** Heatmaps and wavelet power spectrum of influenza virus. A, Time series of monthly age-specific positive rates of A/H3N2, seasonal A/H1N1, A/H1N1pdm09, Victoria, and Yamagata. B, Wavelet power spectrum of the monthly positive rates of influenza subtypes and lineages. Black lines highlight periodicities that reach statistical significance of 95% based on 1000 Monte Carlo simulation. The region outside the white-curved cone indicate the presence of edge effects. The power values were shown in the panel on the right. Time series have been square-root transformed. (EPS 2380 kb)

Additional file 2: **Table S1.** Number of cases of influenza like illness (ILI) and influenza virus-positive specimens by season in Chengdu, 2006/07–2015/16. (XLSX 15 kb)

## Abbreviations

HI test: Hemagglutination inhibition
ILI: Influenza-like illness
MDCK cell: Madin-Darby canine kidney cell
RT-PCR: Real-time reverse transcription PCR
SPF: Specific pathogen free
TIV: Trivalent inactivated influenza vaccine

## References

[1] Simonsen L (1999). The global impact of influenza on morbidity and mortality. Vaccine.

[2] Lipsitch M, Riley S, Cauchemez S (2009). Managing and reducing uncertainty in an emerging influenza pandemic. N Engl J Med.

[3] Lipsitch M, Viboud C (2009). Influenza seasonality: lifting the fog. Proc Natl Acad Sci U S A.

[4] Tamerius J, Nelson MI, Zhou SZ (2011). Global influenza seasonality: reconciling patterns across temperate and tropical. Environ Health Perspect.

[5] Azziz Baumgartner E, Dao CN, Nasreen S (2012). Seasonality, timing, and climate drivers of influenza activity worldwide. J Infect Dis.

[6] Caini S, Andrade W, Badur S (2016). Temporal patterns of influenza a and B in tropical and temperate countries-what are the lessons for influenza vaccination. PLoS One.

[7] Caini S, Andrade W, Badur S (2016). Divergent seasonal patterns of influenza types a and B across latitude gradient in tropical Asia. Influenza Other Respir Viruses.

[8] Fraser C, Donnelly CA, Cauchemez S (2009). Pandemic potential of a strain of influenza a (H1N1): early findings. Science.

[9] Garten RJ, Davis CT, Russell CA (2009). Antigenic and genetic characteristics of swine-origin 2009 a(H1N1) influenza viruses circulating in humans. Science.

[10] Nelson MI, Tan Y, Ghedin E (2011). Phylogeography of the spring and fall waves of the H1N1/2009 pandemic influenza virus in the United States. J Virol.

[11] Baillie GJ, Galiano M, Agapow PM (2012). Evolutionary dynamics of local pandemic H1N1/2009 influenza virus lineages revealed by whole-genome analysis. J Virol.

[12] Yang L, Chan KH, Suen LK (2015). Impact of the 2009 H1N1 pandemic on age-specific epidemic curves of other respiratory viruses: a comparison of pre-pandemic, pandemic and post-pandemic periods in a Subtropical City. PLoS One.

[13] Wang W, Pan M, Chang GH (2009). Laboratory confirmation of the first influenza a( H1N1) imported case in mainland China. Bing Du Xue Bao.

[14] NBS Survey Office in Sichuan (2016). Statistical Bureau of Sichuan.

[15] Feng L, Shay DK, Jiang Y (2012). Influenza-associated mortality in temperate and subtropical Chinese cities, 2003–2008. Bull World Health Organ.

[16] The guideline for national influenza surveillance. Chinese Center for Disease Control and Prevention. 2010. http://www.chinacdc.cn/jkzt/crb/lxxgm/jc/200509/t20050908_24127.htm. Accessed 31 May 2018.

[17] Viboud C, Bjornstad ON, Smith DL (2006). Synchrony, waves, and spatial hierarchies in the spread of influenza. Science.

[18] Alonso WJ, Viboud C, Simonsen L (2007). Seasonality of influenza in Brazil: a traveling wave from the Amazon to the subtropics. Am J Epidemiol.

[19] Torrence C, Compo GP (1998). A practical guide to wavelet analysis. Bull Am Meteorol Soc.

[20] Maraun D, Kurths J, Holschneider M (2007). Nonstationary Gaussian processes in wavelet domain: synthesis, Estimation and Significance Testing. Phys Rev E.

[21] Maraun D, Kurths J (2004). Cross wavelet analysis: significance testing and pitfalls. Nonlin Proc Geoph.

[22] Cheng X, Tan Y, He M (2013). Epidemiological dynamics and phylogeography of influenza virus in southern China. J Infect Dis.

[23] Weinberger DM, Krause TG, Mølbak K (2012). Influenza epidemics in Iceland over 9 decades: changes in timing and synchrony with the United States and Europe. Am J Epidemiol.

[24] Thai PQ, Choisy M, Duong TN (2015). Seasonality of absolute humidity explains seasonality of influenza-like illness in Vietnam. Epidemics.

[25] Hua S, Li X, Liu M (2015). Antigenic variation of the human influenza a (H3N2) virus during the 2014-2015 winter season. Sci China Life Sci.

[26] WHO (2014). Recommended composition of influenza virus vaccines for use in the 2014–2015 northern hemisphere influenza season. Wkly Epidemiol Rec.

[27] Yang L, Wong CM, Lau EH (2008). Synchrony of clinical and laboratory surveillance for influenza in Hong Kong. PLoS One.

[28] Wu Z, Sun X, Chu Y (2016). Coherence of influenza surveillance data across different sources and age groups, Beijing, China, 2008-2015. PLoS One.

[29] Liu T, Li Z, Lin Y (2016). Dynamic patterns of circulating influenza virus from 2005 to 2012 in Shandong Province. China Arch Virol.

[30] Lin J, Kang M, Zhong H (2013). Influenza seasonality and predominant subtypes of influenza virus in Guangdong, China, 2004-2012. J Thorac Dis.

[31] Qi L, Xiong Y, Xiao B (2016). Epidemiological and Virological characteristics of influenza in Chongqing, China, 2011-2015. PLoS One.

[32] He D, Lui R, Wang L (2015). Global spatio-temporal patterns of influenza in the post-pandemic era. Sci Rep.

[33] Yu H, Alonso WJ, Feng L (2013). Characterization of regional influenza seasonality patterns in China and implications for vaccination strategies: Spatio-temporal modeling of surveillance data. PLoS Med.

[34] Rambaut A, Pybus OG, Nelson MI (2008). The genomic and epidemiological dynamics of human influenza a virus. Nature.

[35] Bedford T, Riley S, Barr IG (2015). Global circulation patterns of seasonal influenza viruses vary with antigenic drift. Nature.

[36] Borja-Aburto VH, Chowell G, Viboud C (2012). Epidemiological characterization of a fourth wave of pandemic a/H1N1 influenza in Mexico, winter 2011–2012: age shift and severity. Arch Med Res.

[37] Mossong J, Hens N, Jit M (2008). Social contacts and mixing patterns relevant to the spread of infectious diseases. PLoS Med.

[38] Glass LM, Glass RJ (2008). Social contact networks for the spread of pandemic influenza in children and teenagers. BMC Public Health.

[39] Wu JT, Cowling BJ, Lau EH (2010). School closure and mitigation of pandemic (H1N1) 2009. Hong Kong Emerg Infect Dis.

[40] Yang L, Chan KH, Suen LK (2015). Age-specific epidemic waves of influenza and respiratory syncytial virus in a subtropical city. Sci Rep.

[41] Feng L, Mounts AW, Feng Y (2010). Seasonal influenza vaccine supply and target vaccinated population in China, 2004–2009. Vaccine.

[42] Gupta V, Dawood FS, Muangchana C (2012). Influenza vaccination guidelines and vaccine sales in Southeast Asia: 2008–2011. PLoS One.

[43] Navarro-Marí JM, Pérez-Ruiz M, Cantudo-Muñoz P (2005). Influenza-like illness criteria were poorly related to laboratory-confirmed influenza in a sentinel surveillance study. J Clin Epidemiol.

