# Correction to: Temporal patterns of influenza A subtypes and B lineages across age in a subtropical city, during pre-pandemic, pandemic, and post-pandemic seasons

**DOI:** 10.1186/s12879-019-3807-8

**Published:** 2019-03-28

**Authors:** Linlin Zhou, Huiping Yang, Yu Kuang, Tianshu Li, Jianan Xu, Shuang Li, Ting Huang, Chuan Wang, Wanyi Li, Mingyuan Li, Shusen He, Ming Pan

**Affiliations:** 1 Department of Microbiology, West China School of Basic Medical Sciences & Forensic Medicine, Sichuan University, Chengdu, 610041 China; 2 Sichuan Center for Disease Control and Prevention, No. 6 Zhongxue Road, Wuhou District, Chengdu, 610041 Sichuan People’s Republic of China; 3 Department of Medical Technology, West China School of Public Health, Sichuan University, Chengdu, 610041 China

**Correction to: BMC Infectious Diseases (2019) 19:89**

**https://doi.org/10.1186/s12879-019-3689-9**

Following publication of the original article [1], the author reported an error in the title: ‘Temporal patterns of influenza a subtypes and B lineages across age in a subtropical city, during pre-pandemic, pandemic, and post-pandemic seasons’ should be replace with ‘Temporal patterns of influenza A subtypes and B lineages across age in a subtropical city, during pre-pandemic, pandemic, and post-pandemic seasons’.

It has been corrected in the original article as well.

The publisher apologizes for any inconvenience caused by this error.

## References

[1] Zhou et al. BMC Infect Dis (2019) 19:89, 10.1186/s12879-019-3689-9.

